# Rapid behavioural gregarization in the desert locust, *Schistocerca gregaria* entails synchronous changes in both activity and attraction to conspecifics

**DOI:** 10.1016/j.jinsphys.2014.04.004

**Published:** 2014-06

**Authors:** Stephen M. Rogers, Darron A. Cullen, Michael L. Anstey, Malcolm Burrows, Emma Despland, Tim Dodgson, Tom Matheson, Swidbert R. Ott, Katja Stettin, Gregory A. Sword, Stephen J. Simpson

**Affiliations:** a School of Biological Sciences, A08 – Heydon-Laurence Building, and the Charles Perkins Centre, University of Sydney, NSW 2006, Australia; b Department of Zoology, University of Cambridge, Downing St, Cambridge CB2 3EJ, UK; c Department of Biology, University of Leicester, University Road, Leicester LE1 7RH, UK; d Concordia University, 7141 Sherbrooke St. West, SP-375-09, Montreal H4B 1R6, QC, Canada; e Department of Entomology, Texas A&M University, TAMU 2475, College Station, TX 77843-2475, USA

**Keywords:** Phase change, Density dependent polyphenism, Phenotypic plasticity, Behavioural plasticity, Group attraction, Serotonin

## Abstract

Desert Locusts can change reversibly between solitarious and gregarious phases, which differ considerably in behaviour, morphology and physiology. The two phases show many behavioural differences including both overall levels of activity and the degree to which they are attracted or repulsed by conspecifics. Solitarious locusts perform infrequent bouts of locomotion characterised by a slow walking pace, groom infrequently and actively avoid other locusts. Gregarious locusts are highly active with a rapid walking pace, groom frequently and are attracted to conspecifics forming cohesive migratory bands as nymphs and/or flying swarms as adults. The sole factor driving the onset of gregarization is the presence of conspecifics. In several previous studies concerned with the mechanism underlying this transformation we have used an aggregate measure of behavioural phase state, *P*_greg_, derived from logistic regression analysis, which combines and weights several behavioural variables to characterise solitarious and gregarious behaviour. Using this approach we have analysed the time course of behavioural change, the stimuli that induce gregarization and the key role of serotonin in mediating the transformation. Following a recent critique that suggested that using *P*_greg_ may confound changes in general activity with genuine gregarization we have performed a meta-analysis examining the time course of change in the individual behaviours that we use to generate *P*_greg_. We show that the forced crowding of solitarious locusts, tactile stimulation of the hind femora, and the short-term application of serotonin each induce concerted changes in not only locomotion-related variables but also grooming frequency and attraction to other locusts towards those characteristic of long-term gregarious locusts. This extensive meta-analysis supports and extends our previous conclusions that solitarious locusts undergo a rapid behavioural gregarization upon receiving appropriate stimulation for a few hours that is mediated by serotonin, at the end of which their behaviour is largely indistinguishable from locusts that have been in the gregarious phase their entire lives.

## Highlights

• Desert Locusts can transform between solitarious and gregarious phases.
• Stimuli from conspecifics cause rapid behavioural gregarization in just 4 h.
• Gregarization entails synchronous changes in several discrete behaviours.
• Overall activity and attraction to other locusts change at the same rate.
• Serotonin causes changes in both attraction and locomotion.

## Graphical abstract

**Figure:**
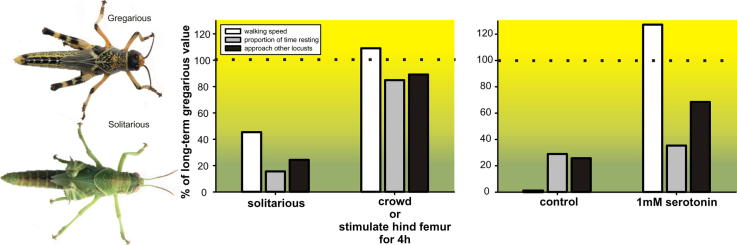

## 1 Introduction

Desert Locusts, *Schistocerca gregaria*, display a dramatic polyphenism, being able to transform reversibly between two extreme forms or phases that differ considerably in many aspects of their biology including behaviour, physiology and morphology, (Uvarov, 1966, 1977; Roessingh et al., 1993; Simpson et al., 1999; Tawfik et al., 1999; Hoste et al., 2002; Rogers et al., 2004; Pener and Simpson, 2009; Harano et al., 2012). Phase change is driven by fluctuations in population density: when population density is low, locusts occur in the solitarious phase, but increasing numbers and the concentration of locusts onto scarce resources triggers the transformation to the gregarious phase. The process of gregarization is initiated and maintained by stimuli from other locusts, but it is a process that occurs over many different time scales, from hours to generations. Behaviour is the most labile aspect of the phenotype and is in many ways the enabling step for the entire process since solitarious locusts avoid other locusts, all other circumstances being equal, whereas gregarious locusts are mutually attracted to each other, leading to the formation of coherent migratory bands. Within just a few hours of forced contact with other locusts (Roessingh and Simpson, 1994) or receiving appropriate sensory stimulation (Ellis, 1959, 1963; Heifetz et al., 1996; Roessingh et al., 1998; Simpson et al., 1999, 2001; Rogers et al., 2003) key aspects of behaviour are reconfigured away from those typical of solitarious locusts (low activity and repelled by other locusts) to a set of behaviours typical of long-term gregarious locusts (high activity and attracted to other locusts).

Since this behavioural transformation encompasses several discrete behavioural changes, binary logistic regression analysis has been used for many years as a means to characterise the solitarious and gregarious behavioural states in several species of locust (Roessingh et al., 1993, Roessingh and Simpson 1994, Roessingh et al., 1998; Bouaichi et al., 1995, 1996; Islam et al., 1994; Simpson et al., 1999, 2001; Hagele and Simpson, 2000; Rogers et al., 2003; Sword, 2003; Lester et al., 2005; Anstey et al., 2009; Gray et al., 2009; Cullen et al., 2010, 2012; Ma et al., 2011; Ott et al., 2012). Logistic regression is a useful method of classifying locust behaviour as it encapsulates several distinct activities and condenses them into a single metric of phase state. This metric, called *P*_greg_, is the probability that a locust of unknown behavioural phase state, for example one that has been subjected to some experimental treatment, should be considered as belonging to a model population of known gregarious or solitarious locusts previously observed under the same conditions. These model populations consist of approximately 100 independent observations of locusts that have been kept in isolation for three generations and 100 observations of locusts kept in high-density culture for many generations, providing a substantial baseline for the expected behaviour of each phase. A *P*_greg_ of 0 indicates that the locust’s behaviour is indistinguishable from the solitariously-reared model population, whereas a *P*_greg_ of 1 indicates fully gregarious behaviour. Transitional states between the two extremes can be quantified so that a direct link can be made between changes in the central nervous system (CNS) and behaviour. This in turn gives users a way to assess quantitatively the effects of pharmacological or other treatments on the propensity of locusts to gregarize or solitarize.

*P*_greg_ is derived from a logistic algorithm where *P*_greg_ = e*^η^*/(1 + e*^η^*), and *η* = β_0_ + β_1_x_1_ + β_2_x_2_ + … + β*_k_*x*_k_* and where *η* is a weighted (β*_k_*) sum of *k* individual behavioural measurements (x*_k_*) that are found to be strong predictors of phase state during the initial building of the model. The number and precise type of behaviours used to calculate *P*_greg_ have varied over time with different experimenters, but have always consisted of a mix of locomotory-related behaviours, indicators of attraction to or repulsion from other locusts, and discrete behaviours. In recent years we have settled on a model based on measurements of four different parameters: (1) how fast the locusts walk (walk speed; locomotion related); (2) how much time as a proportion of the total observation time locusts spend motionless (rest time fraction; locomotion-related); (3) how much time locusts spend in the 25% of the arena closest to a group of gregarious locusts kept behind a clear perforated partition at one end (time at the stimulus end; indicator of attraction); and (4) the frequency with which locusts groom over the entire observation period (total grooming frequency; discrete behaviour). Using these four behaviours in a logistic regression analysis provides a robust separation of known solitarious and gregarious locusts, with typically ⩾90% of locusts from the model-building population being correctly classified on the basis of their behaviour during the ten minute assay.

The logistic regression approach allows individual experimental locusts to be assigned to either the solitarious or gregarious phase with a defined probability, increasing the statistical power available to compare the effects of experimental treatments. Using this technique we have determined the time course of behavioural change (Roessingh and Simpson, 1994; Anstey et al., 2009), identified the stimuli that trigger the transformation (Roessingh et al., 1998; Simpson et al., 1999, 2001; Rogers et al., 2003) and established the key roles of serotonin and Protein Kinase A in mediating behavioural gregarization (Anstey et al., 2009; Ott et al., 2012). Other methods of classifying behaviour based on aggregation of groups of locusts (Ellis, 1959; Gillett, 1973; Heifetz et al., 1996) do not allow this individual determination of phase state, as the outcomes of those assays are determined at the level of the group.

The use of *P*_greg_ as a single metric of behavioural phase state combines changes in several discrete behaviours. As such, it does not allow an independent assessment of how changes in these individual behaviours contribute to the overall change in *P*_greg_ value. Although by design logistic regression analysis is robust to changes in single behaviours, it is theoretically possible for changes in one or a few behaviours to account for changes in *P*_greg_ and that, rather than measuring a coherent change in overall phase state, the value is responding to successive changes in individual and non-linked behaviours. A recent paper by Tanaka and Nishide (2013) posits that the rapid onset of behavioural gregarization in just a few hours that has been consistently observed in studies by several groups since the 1950s is driven mostly or entirely by changes in activity rather than changes in the attraction/repulsion to other locusts. In other words, they argue that previous studies using logistic regression to quantify behavioural phase state may not have measured true behavioural gregarization that encompasses both activity and attraction to conspecifics. Tanaka and Nishide (2013) also suggest that genuine behavioural gregarization is a much slower process taking days rather than hours, based on a metric that purely considers the proximity of a test locust to a group of stimulus locusts kept in a clear container as a measure of attraction. Further, Tanaka and Nishide (2013) go on to question the role of serotonin (Anstey et al., 2009; Ott et al., 2012) in initiating the expression of attraction-related behaviours as part of gregarization in *S. gregaria*.

Here we present a full meta-analysis of the underlying univariate data used in logistic regression analyses of locust behavioural phase change in order to show explicitly how different behaviours are affected by gregarizing stimuli and what effect serotonin has on the different behavioural components of *P*_greg_. The principal aim of this analysis is to demonstrate explicitly how each of the behaviours used to generate *P*_greg_ change during the rapid initial process of gregarization following either forced crowding with other locusts or mechanosensory stimulation of the hind femora. We also present a detailed break-down of how serotonin and its antagonists affect individual behaviours. These analyses show that the rapid gregarization that we have consistently observed is the consequence of coherent changes in both activity and attraction-related behaviours and that *P*_greg_ does indeed provide a robust measure of phase state in *S.* *gregaria*.

## 2 Materials and methods

The data presented in this meta-analysis were derived from work previously published in Simpson et al. (2001), Rogers et al. (2003) and Anstey et al. (2009). The assay arena used to assess behavioural phase consists of a rectangular space 41 cm long × 31 cm wide × 10 cm high with two clear perforated partitions at each end, behind each of which is a chamber 8 cm long × 31 cm wide × 10 cm high (Roessingh et al., 1993; Simpson et al., 1999; Cullen et al., 2012). A ‘stimulus group’ of 30 long-term gregarious locusts is placed in one of these chambers so that a locust in the main arena can see and smell this group and is able to approach or avoid it, while the other chamber is left empty. An experimental locust is introduced into the arena via a hole in the centre of its floor and detailed observations of its behaviour, including continuous measures of its location, velocity and trajectory as well as discrete events such as head, antennal movements and leg movements are made for the next 500 s.

**Fig. 1:**
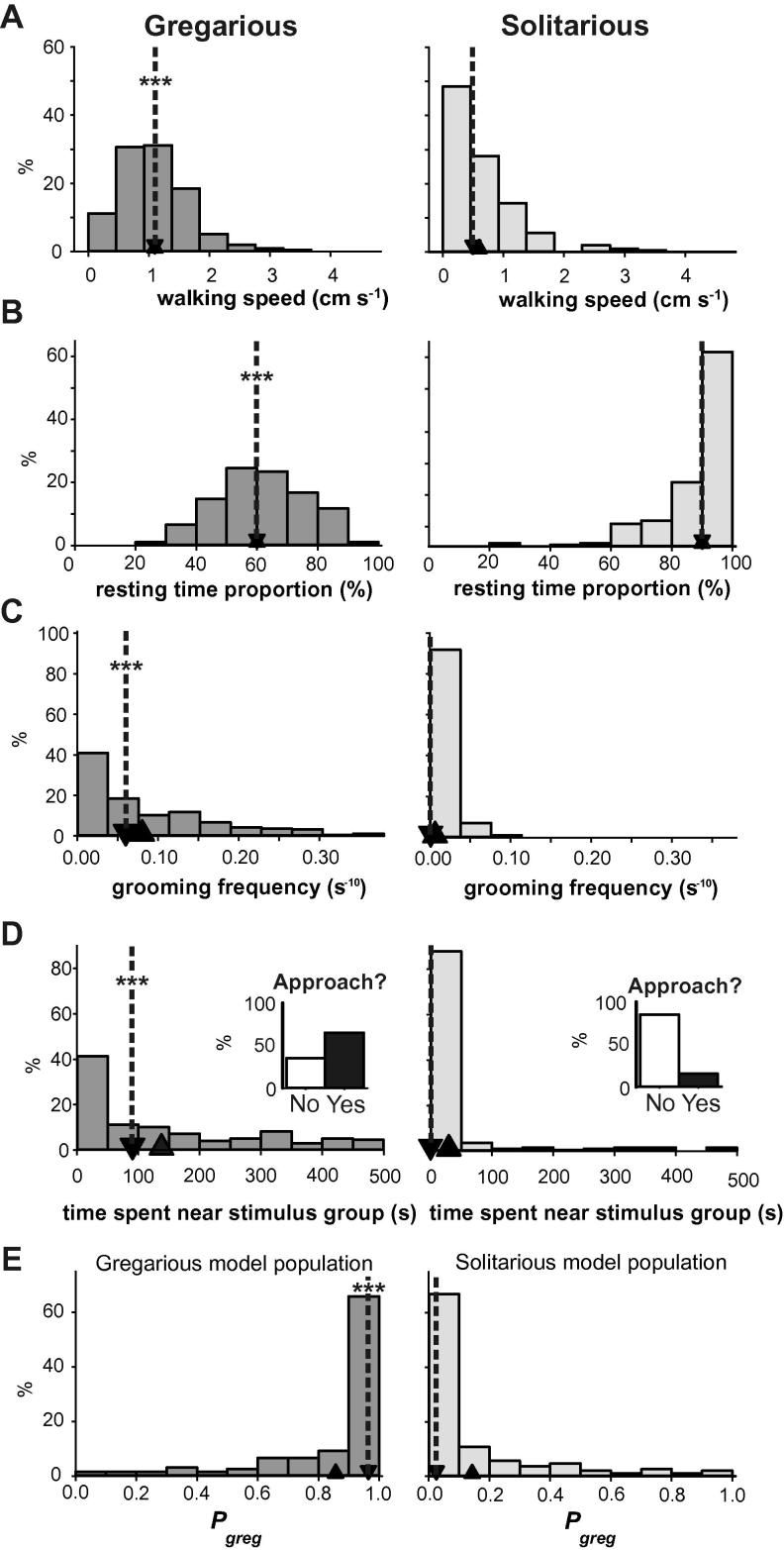
The behaviours used to generate the logistic regression model of behavioural phase in *Schistocerca gregaria* based on their performance in the test arena. Data are from 196 locusts kept under crowded conditions for many generations (gregarious) and 196 locusts kept isolated for three generations (solitarious). The behaviours used in the model are (A) walking speed, (B) the resting time fraction, (C) the frequency of grooming and (D) the time spent in the quadrant of the arena next to a group of 50 gregarious locusts kept behind a clear partition. The inset graphs show the percentage of locusts approaching the stimulus group at least once during the 500 s assay period. (E) These behaviours when combined using the logistic equation allow these two groups to be separated with a high degree of confidence (*P*_greg_). Down-pointing arrow heads with dotted lines show the location of the median value; up-pointing arrow heads show the location of the mean value for each data set. ^∗∗∗^Indicates difference between solitarious and gregarious populations < 0.001; See also Tables 1 and 2.

For this meta-analysis the data set used to generate the logistic regression models in Simpson et al., 2001 and Rogers et al. (2003) was combined with that used in Anstey et al. (2009) to give a base number of 196 each of known long-term gregarious locusts (reared in dense culture for 20+ years) and locusts reared in visual, tactile and olfactory isolation for three generations (hereafter referred to as ‘solitarious’). A new logistic regression model was fitted to these data using the same behavioural covariates as in Anstey et al., 2009 (Table 1), namely: walking speed (Fig. 1A), fraction of time spent motionless (Fig. 1B), time spent in the 25% area of the arena next to the stimulus group of locusts (Fig. 1C) and frequency of grooming (Fig. 1D). When applying *P*_greg_ = 0.5 as the cut-off, this model correctly classified 90.8% of gregarious locusts and 91.3% of solitarious locusts of the model populations, with 65.8% of gregarious locusts having *P*_greg_ values > 0.9 (median 0.97; interquartile range, IQR, 0.8–1.0) and 66.8% of solitarious locusts having a *P*_greg_ < 0.1 (median 0.04; IQR 0.01–0.15; Fig. 1E).

Full details of how locusts were crowded or given mechanosensory stimulation to induce behavioural gregarization are given in the original publications (Simpson et al. (2001), Rogers et al. (2003), Anstey et al. (2009)). In brief: solitarious locusts were crowded by placing them in plastic terrariums (21 × 13 × 17 cm) together with 50 final larval instar long-term gregarious locusts. Groups of approximately 10 solitarious locusts were crowded at a time, numbered with a marker pen and with their entrance to the crowding cage staggered at 10 min intervals so that crowding times were precisely 1, 2 or 4 h. Locusts given mechanosensory stimulation to the hind femora were placed in small acrylic boxes (8 × 6 × 10.5 cm) with open mesh ends through which a small paint brush could be pushed to tickle a hind femur for 5 s at 1–2 min intervals for either 1, 2 or 4 h. Control locusts were handled in a similar manner but kept in cages on their own without stimulation. Details of the methods used to administer serotonin, its precursor and agonists are given in Anstey et al., 2009, and briefly recapped in the relevant results section below.

The logistic regression model and all statistical tests were performed using IBM SPSS statistics (version 21). Simulations of the effect of systematically altering one or two behavioural covariates on *P*_greg_ were performed in Sigma Plot (version 11, Systat Software Inc.) using the logistic equation and constants generated by the population model (Table 1).

## 3 Results

### 3.1 The model populations

From the individual behavioural variables that define the model (Table 2) it is clear that long-term gregarious locusts were more active than long-term solitarious locusts, spending only 61% (51–72%) (Median (interquartile range)) of their time motionless as opposed to 94% (84–99%) for solitarious locusts. Gregarious locusts also had a higher walking speed (1.11 cm s^−1^; 0.72–1.40 cm s^−1^) than solitarious locusts (0.53 cm s^−1^; 0–0.94 cm s^−1^). These two variables are related to general activity, but gregarious locusts also groomed more often (0.006 s^−1^; 0–0.014 s^−1^), whereas it was a very rare occurrence for solitarious locusts (0 s^−1^; 0–0 s^−1^). Gregarious locusts were also far more likely to spend time in the quadrant of the arena closest to the stimulus group of locusts (90.5 s; 0–259 s) whereas the great majority of solitarious locusts avoided this area entirely (0 s; 0–0 s). If these data are reduced to a simple binary measure of whether or not a locust approached the stimulus group, then 64.8% of gregarious locusts approached the other locusts, whereas only 15.4% of solitarious locusts did so (*χ*^2^ = 208, *P* < 0.001; inserts in Fig. 1C).

### 3.2 The effect of crowding

**Fig. 2:**
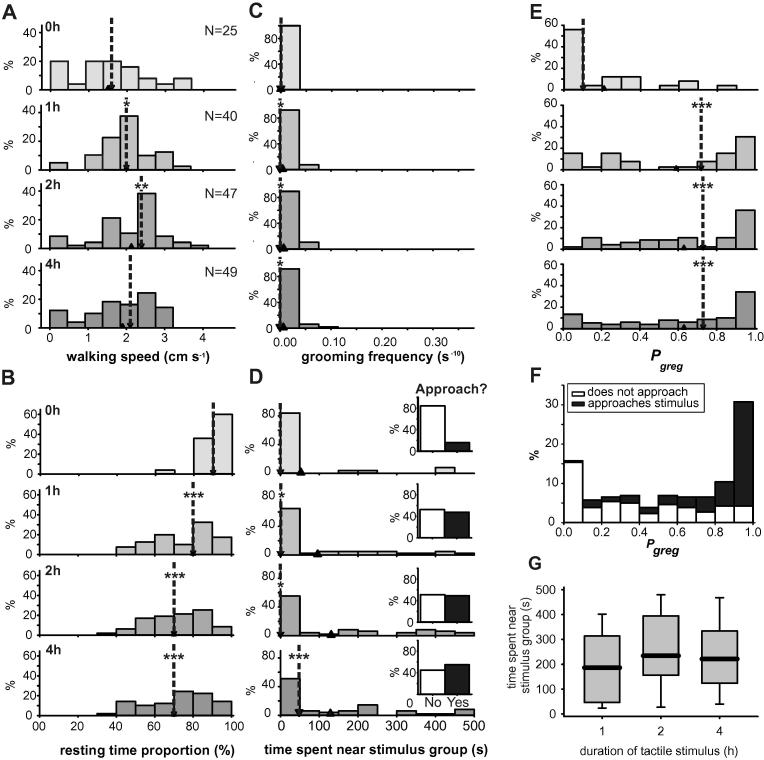
The effect of crowding solitarious locusts for 0 h (control), 1, 2 or 4 h on the behavioural characteristics used to generate *P*_greg_. (A) Walking speed, (B) percentage of time spent motionless, (C) grooming frequency, (D) time spent in the quadrant of the arena adjacent to a stimulus group of gregarious locusts. The inset graphs show the percentage of locusts approaching the stimulus group at least once (black) or not at all (white) during the 500 s assay period. (E) The distribution of *P*_greg_ as derived from these behavioural parameters. Down-pointing arrow heads with dotted lines show the location of the median value; up-pointing arrow heads show the location of the mean value for each data set. (F) The distribution of *P*_greg_ across the entire experiment, split according to whether locusts approached the stimulus group (black) or avoided it (white). (G) Time spent near the stimulus group, including only those locusts that made at least one approach during the assay. Graph show medians (black line), interquartile ranges (boxes) and 90% ranges (whiskers). ^∗^Indicates difference between control and crowded groups *P* < 0.05; ^∗∗^indicates *P* < 0.01; ^∗∗∗^*P* < 0.001; See also Table 3.

Crowding induced a change in all four behavioural characters, starting after just 1 h and continuing up to 4 h of crowding (Fig. 2; Table 3). This is reflected by a matching shift in *P*_greg_ towards the gregarious end of the scale (Fig. 2E) so that after 4 h of crowding the median *P*_greg_ was 0.73 (0.32–0.95). In these experiments the walking speed was unusually high even in the un-crowded controls (1.6 cm s^−1^; 1.1–2.12 cm s^−1^; Fig. 2A), but this by itself was insufficient to induce a marked change in *P*_greg_ away from solitarious values, as all other behavioural characteristics were typical of the solitarious model population in the control animals. Walking speed increased over the first 2 h of crowding (up to 2.40 cm s^−1^; 1.66–2.73 cm s^−1^) before falling back at 4 h to 2.14 cm s^−1^ (1.4–2.53 cm s^−1^).

The proportion of time spent motionless (Fig. 2B) decreased from 93% (89–99%) in the controls down to 81% (63–87%) after 1 h of crowding before dropping to 74% (58–86%) after 4 h. This was still significantly less active than the long-term gregarious model population (61% (51–72%); Mann–Whitney *U* = 749, *P* = 0.001, *N* = 49 crowded and 49 long-term gregarious locusts randomly selected from the model population). The frequency of grooming was significantly greater after 1, 2 and 4 h of crowding than in un-crowded controls (Table 3). Grooming was, however, still less frequent after 4 h of crowding than in long-term gregarious locusts (Mann–Whitney *U* = 383, *P* < 0.001, *N* = 49 crowded and 49 long-term gregarious locusts randomly selected from the model population) and the median frequency did not increase above 0 (Fig. 2C).

The time spent near the stimulus group increased from a median of 0 s (0–0 s) in the control group up to 48 s (0–224 s) after 4 h of crowding (Fig. 2D), which was statistically indistinguishable from long-term gregarious locusts (Mann–Whitney *U* = 11,143, *P* = 0.673, *N* = 49 crowded and 49 long-term gregarious locusts randomly selected from the model population). Considered as a binary approach/avoid statistic over the 500 s observation time, the proportion of locusts approaching the stimulus group at some point during the observation rose from 16% in the solitarious controls to 47.5% after just 1 h of crowding. After 4 h of crowding the majority of locusts (55%) were approaching the other locusts in the arena. Again, this is not statistically different from the 65% of long-term gregarious locusts approaching the stimulus group in the model population (*χ*^2^ = 2.252, *P* = 0.133, *N* = 49). In Fig. 2F the distribution of *P*_greg_ across the entire experiment (0–4 h) is split according to whether the locusts approached the stimulus group (black) or not (white). It was a rare occurrence for locusts with high *P*_greg_ not to approach the stimulus group (only 16.7% of locusts where *P*_greg_ > 0.9) whereas not a single locust with low *P*_greg_ (<0.1) entered the quadrant nearest other locusts. Locusts that did enter this quadrant spent 221 s (124–334 s) in it, equating to 17–73% of their total time in the arena. The time spent next to the stimulus group in these animals did not significantly increase with the duration of crowding (Fig. 2G; Kruskal–Wallis test, *χ*^2^ = 2.06, *P* = 0.357).

### 3.3 The effect of mechanosensory stimulation

**Fig. 3:**
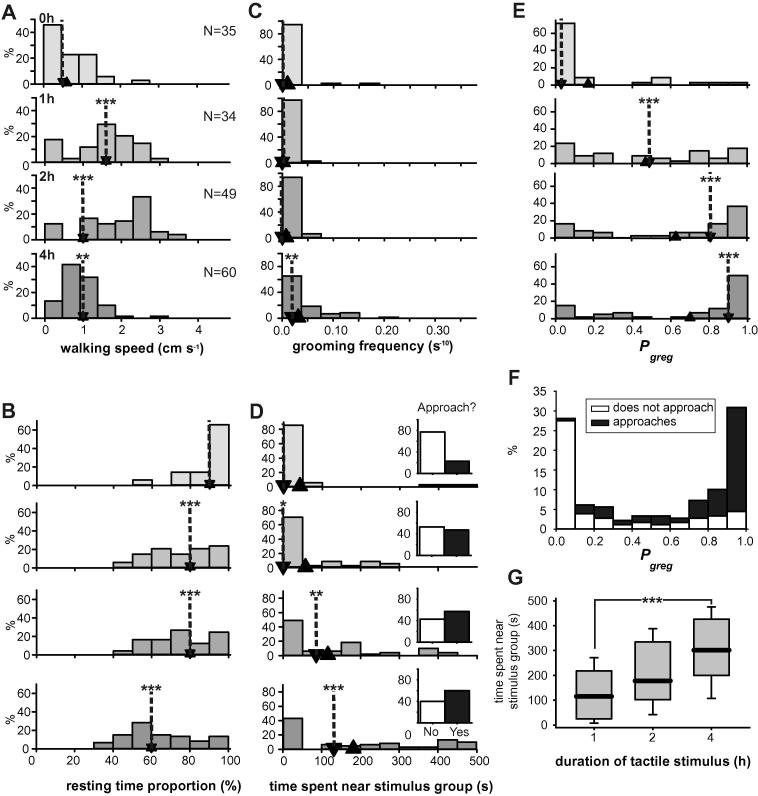
The effect of tactile stimuli directed to the hind femora on the behaviour of solitarious locusts after 0 h (control), 1, 2 or 4 h stimulation. (A) Walking speed, (B) proportion of time spent resting, (C) grooming frequency, (D) time spent in the quadrant of the arena adjacent to a stimulus group of gregarious locusts. The inset graphs show the percentage of locusts approaching the stimulus group at least once (black) or not at all (white) during the 500 s assay period. (E) The distribution of *P*_greg_ as derived from these behavioural parameters. Down-pointing arrow heads with dotted lines show the location of the median value; up-pointing arrow heads show the location of the mean value for each data set. (F) The distribution of *P*_greg_ across the entire experiment, split according to whether locusts approached the stimulus group (black) or avoided it (white). (G) Time spent near the stimulus group, including only those locusts that made at least one approach during the assay. Graph show medians (black line), interquartile ranges (boxes) and 90% ranges (whiskers). ^∗^Indicates difference between control and tickled groups *P* < 0.05; ^∗∗^indicates *P* < 0.01; ^∗∗∗^*P* < 0.001; See also Table 4.

Repeatedly stroking the hind femora induced changes in all four behavioural characteristics used to generate *P*_greg_ (Fig. 3; Table 4), with three of the four behaviours becoming significantly different from the controls after just 1 h of treatment; only grooming frequency took 4 h to become significantly different (Table 4). In these experiments the control locusts had walking speeds similar to the solitarious model population (0.55 cm s^−1^; 0–1.1 cm s^−1^), but as with the crowded locusts there was an initial surge in walking speed to values well above those typical of long-term gregarious locusts, up to 1.62 cm s^−1^ (1.24–2.20 cm s^−1^; Fig. 3A). After 4 h of stimulation, however, the median velocity had fallen back to 0.95 cm s^−1^ (0.7–1.21 cm s^−1^), slightly lower than those of the gregarious model population (Mann–Whitney *U* = 1390, *P* = 0.031, *N* = 60 stimulated and 60 long-term gregarious locusts randomly selected from the model population).

The proportion of time spent motionless decreased steadily over the course of the experiments (Fig. 3B), from 94% (86–97%) in controls down to 77% (61–90%) after 1 h stimulation and 60% (53–78%) after 4 h stimulation, at which point it was indistinguishable from the long-term gregarious model population (Mann–Whitney *U* = 1734, *P* = 0.727, *N* = 60 stimulated and 60 long-term gregarious locusts randomly selected from the model population). After 4 h of stimulation, grooming frequency had increased from control levels to 0.002 s^−1^ (0–0.004 s^−1^), but was still lower than in the long-term gregarious population (Fig. 3C; Mann–Whitney *U* = 1074, *P* < 0.001, *N* = 60 stimulated and 60 long-term gregarious locusts randomly selected from the model population).

The amount of time spent next to the stimulus group increased with the duration of mechanosensory stimulation (Fig. 3D), rising from a median of 0 s (0–0 s) in the controls and after 1 h (0; 0–110 s), to 85 s (0–189 s) after 2 h, and reaching 131 s (0–387 s) after 4 h. This latter group was statistically indistinguishable from the long-term gregarious model population (Mann–Whitney *U* = 1717, *P* = 0.655, *N* = 60 stimulated and 60 long-term gregarious locusts randomly selected from the model population). If instead of using the duration of proximity to other locusts, a simple binary approach/avoid is considered (insets Fig. 3D) then only 23% of control locusts approached the stimulus group in the arena, rising to 47% after just 1 h of mechanosensory stimulation and reaching 60% after 4 h. Again, after 4 h the proportion of locusts approaching the stimulus group is statistically indistinguishable from the 65% approaching in the long-term gregarious model population (*χ*^2^ = 0.659, *P* = 0.417, *N* = 60).

The changes in these individual behaviours are reflected in the progressive increase in *P*_greg_ across the entire experiment (Fig. 3E), increasing from a median of 0.03 (0.01–0.17) in controls to 0.49 (0.15–0.79) after 1 h, to 0.81 (0.26–0.93) after 2 h before reaching 0.9 (0.37–0.98) after 4 h. If *P*_greg_ across the whole experiment is plotted, split according to whether locusts approached the stimulus group or not (Fig. 3F), a pattern similar to that of the crowding experiments is revealed; only 14.5% of locusts with *P*_greg_ > 0.9 failed to approach the stimulus group whereas 98% of locusts with *P*_greg_ < 0.1 avoided the other locusts. In the locusts that approached the stimulus group, the time spent near it increased steadily with the duration of the tactile stimulation (Fig. 3G), rising from 66 s (22–400 s) in controls to 116 s (28–216 s) after 1 h to 301 (201–426 s) after 4 h (Fig. 3G; Kruskal–Wallis test, *χ*^2^ = 19.04, *P* < 0.001).

#### 3.3.1 The effect of serotonin on phase state

**Fig. 4:**
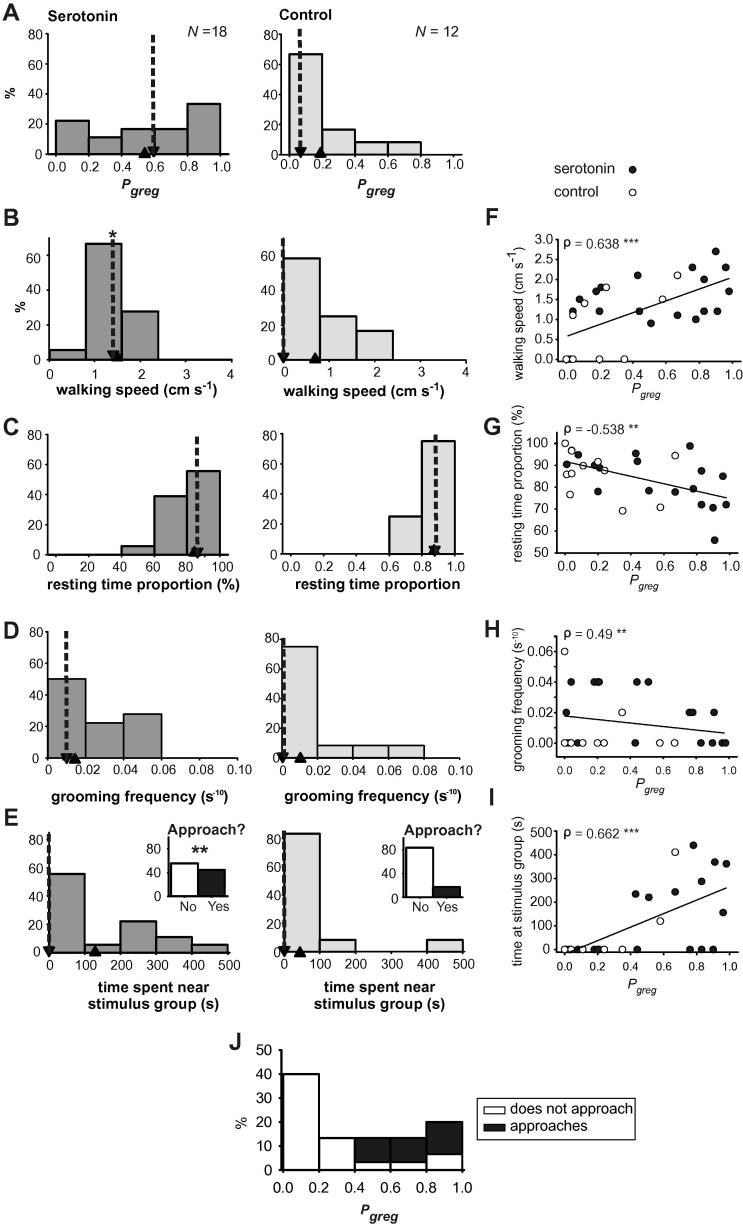
The effect of serotonin applied topically to the thoracic ganglia on the behaviours used to generate *P*_greg_ (A). These were walking speed (B), the proportion of time spent resting (C), the grooming frequency (D) and time spent in the quadrant of the arena adjacent to a stimulus group of gregarious locusts (E). The inset graphs show the percentage of locusts approaching the stimulus group at least once during the 500 s assay period. Down-pointing arrow heads with dotted lines show the location of the median value; up-pointing arrow heads show the location of the mean value for each data set. ^∗^Indicates difference between control and serotonin treated group *P* < 0.05; ^∗∗^indicates *P* < 0.01; See also Table 5. (F–I) The values for each locust of each individual behaviour plotted against the value of *P*_greg_ that were in part obtained from them. Black circles indicate serotonin treated locusts, white circles saline treated controls. Spearman’s correlation coefficients (*ρ*) are given for each graph. Significance of correlations are marked by asterisks: ^∗∗^indicates *P* < 0.01; ^∗∗∗^*P* < 0.001. (J) The distribution of *P*_greg_ across the entire experiment, split according to whether locusts approached the stimulus group (black) or avoided it (white).

We applied 1 mM serotonin solution in saline directly onto the meso- and metathoracic ganglia, which had previously been treated with protease in order to allow the serotonin to cross the blood–brain barrier (Anstey et al., 2009). This treatment or a saline control was applied as a continuous drip for 2 h before the locusts were sealed up and their performance assessed in the arena. There was a marked, but not complete shift towards higher gregariousness in the serotonin-treated locusts (median *P*_greg_ = 0.59; Fig. 4A) compared to controls (median *P*_greg_ = 0.07; Anstey et al., 2009). The histograms of these data show that there was a wide range of *P*_greg_ in the serotonin-treated animals, though the single largest group in was in the *P*_greg_ = 0.8–1 range (Fig. 4A). This distribution of *P*_greg_ (Fig. 4A) suggested that serotonin had variable effectiveness in promoting gregariousness, which is unsurprising given the invasiveness of the procedure necessary to promote its transfusion into the CNS and the need for the locusts to behave in the arena afterwards. The breakdown of the behavioural variables used to generate *P*_greg_ suggests some degree of change in all four characters (Fig. 4B–E), though of itself only walking speed (Fig. 4B) was significantly faster in the serotonin treated locusts, increasing from 0 cm s^−1^ (0–1.4 cm s^−1^) in controls to 1.4 cm s^−1^ (1.2–2 cm s^−1^) in serotonin-treated locusts (Table 5). The median percentage of time spent resting (Fig. 4C) decreased from 89% (81–96%) to 86% (78–92%); the median grooming frequency (Fig. 4D) increased from 0 s^−1^ (0–0.001 s^−1^) to 0.001 s^−1^ (0–0.004 s^−1^), whereas median time spent near the stimulus group (Fig. 4E) remained at 0 s in both control (0–0 s) and treated locusts (0–243 s). If, however, a simple binary statistic of approaching/avoiding the stimulus group is considered, akin to the method used by Tanaka and Nishide, 2013, (insets Fig. 4E), then 44% of locusts approached the stimulus group in the assay after serotonin treatment, whereas only 17% of saline treated locusts did so (*G*-test against an expected 83:17 distribution from the control group; *G* = 7.584, *P* = 0.006), so the serotonin treatment clearly affected the attraction/repulsion behaviour of locusts.

One important question is whether a single behavioural characteristic could be disproportionately affected by the serotonin treatment and cause a pronounced change in *P*_greg_ in the absence of concomitant changes in the other behavioural characteristics, To address this we analysed the relationship between each behavioural character and the values of *P*_greg_ that they contribute towards (Fig. 4F–I). There is a small but progressive increase in walking speed with *P*_greg_ (Fig. 4F; Spearman’s correlation; *ρ* = 0.638, *P* < 0.001). Resting time fraction (Fig. 4G) showed a similar, but negative correlation with *P*_greg_ (Spearman’s *ρ* = −0.538, *P* = 0.002). Grooming frequency (Fig. 4H) was not well described by a linear relationship when plotted against *P*_greg_ but was still significantly correlated (Spearman’s *ρ* = 0.490, *P* = 0.006). No locust with a *P*_greg_ < 0.43 approached the stimulus group (Fig. 4I and J), and the number of locusts that spent time in close proximity to other locusts increased with increasing *P*_greg_ above this value. Conversely, only two locusts with a *P*_greg_ > 0.8 did not approach the stimulus group. Time spent near the stimulus group was significantly correlated with *P*_greg_ (Spearman’s *ρ* = 0.662, *P* < 0.001). Individually all four behaviours used in the model are significantly correlated with the *P*_greg_ values that are obtained in the experimental data from the serotonin injection experiments. If one or more of the behavioural variables were not contributing to the *P*_greg_ values obtained after serotonin (or control) injections then they would not be expected to show any correlation with these *P*_greg_ values. As it is, the strongest correlation with *P*_greg_ was shown by time spent next to the stimulus group, followed by walking speed, resting time fraction and finally grooming frequency. The effect of serotonin on *P*_greg_ is thus clearly not due solely to an increase in locomotory activity, as suggested by Tanaka and Nishide (2013).

**Fig. 5:**
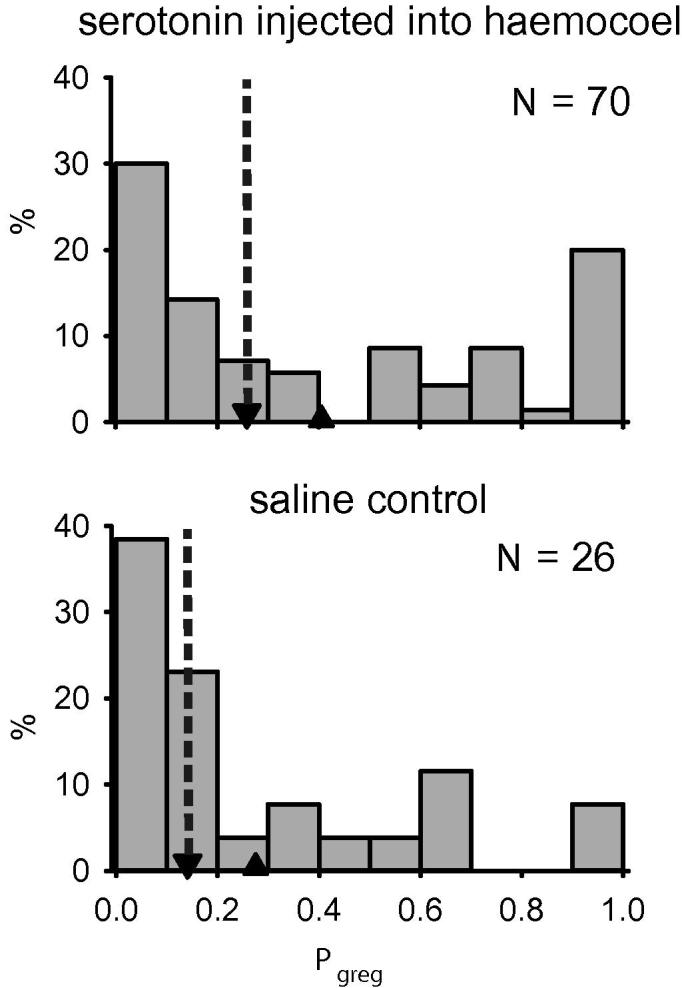
Injecting serotonin into the haemocoel had little effect on behavioural phase state. Histograms showing the distribution of *P*_greg_ in locusts injected with 40 μl of 1 mM serotonin (top) or a saline control (bottom). Down-pointing arrow heads with dotted lines show the location of the median value; up-pointing arrow heads show the location of the mean. See also Table 6.

Prior to developing our method of making the sheath of the CNS permeable to serotonin (Anstey et al. (2009), we tried unsuccessfully (Fig. 5) to induce gregarization by injecting 40 μl of 1 mM serotonin in locust saline into the haemocoel, in a manner similar to the method used by Tanaka and Nishide (2013). These systemic injections produced only a small and non-significant effect on *P*_greg_ or on any of the behaviours used to generate it (Table 6).

**Fig. 6:**
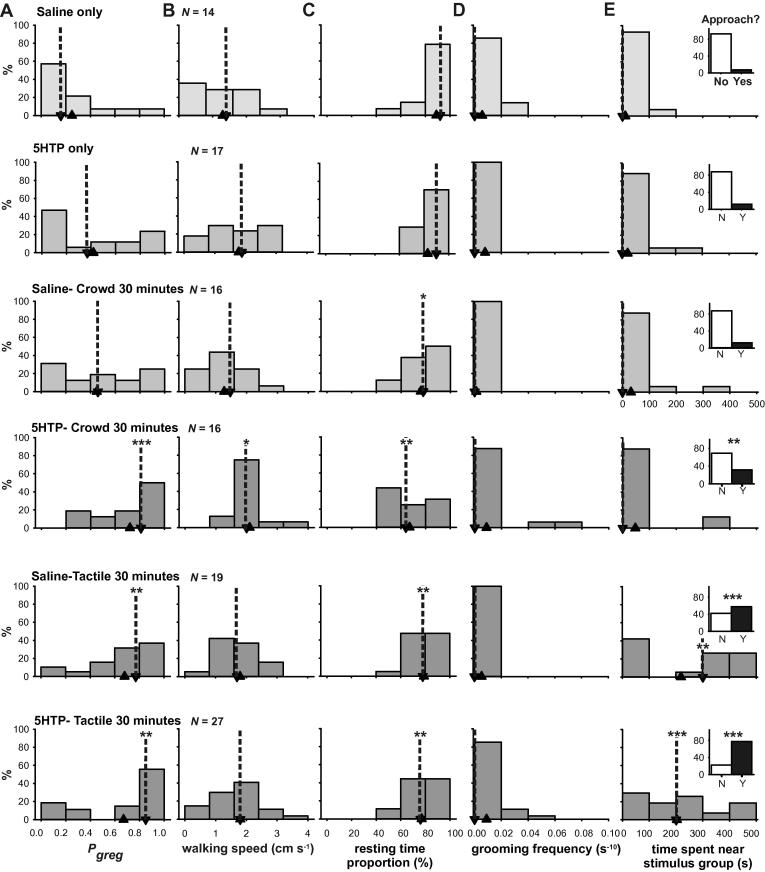
The effect of the serotonin precursor 5 hydroxy tryptophan (5HTP) or a saline control on behavioural phase state, *P*_greg_ (A) and the individual behaviours used to generate it (B-D) either without the application of gregarizing stimuli (top two rows) or combined with brief periods of crowding (middle two rows) or tactile stimulation to the hind femora (bottom two rows). Data are shown as histograms with medians indicated by down-pointing arrow heads with dotted lines and means by up-pointing arrow heads. The behaviours are walking speed (B), resting time proportion (C), grooming frequency (D) or time spent near the stimulus group in the arena (E). ^∗^Indicates difference between control (saline only group) and other treatment is *P* < 0.05; ^∗∗^indicates *P* < 0.01 and ^∗∗∗^*P* < 0.001; See also Table 7. The inset graphs in (E) show the frequency of locusts approaching the stimulus group at least once during the 500 s observation period. Significances of *G*-tests comparing the frequency of approach in the control group with the other treatments are marked ^∗∗^*P* < 0.01 and ^∗∗∗^*P* < 0.001; see also Table 8.

The serotonin precursor molecule 5-hydroxy tryptophan (5HTP) had a significant effect of facilitating gregarization when injected into the haemocoel (40 μl of 10 mM concentration in locust saline) 30 min prior to either 30 min of crowding or mechanosensory stimulation of a hind femur (Anstey et al., 2009). Fig. 6 shows a breakdown into the effects of this treatment on the individual behavioural characters used to generate *P*_greg_ in these experiments. Across the entire experiment there was a significant effect of treatment (Table 7) on the proportion of time spent resting (Fig. 6C) and the time spent near the stimulus group (Fig. 6E). As reported in Anstey et al. (2009), administering 5HTP without subsequent sensory stimulation or crowding control-injected locusts for 30 min had only a weak effect on *P*_greg_; whereas 30 min of tactile stimulation alone was sufficient to cause a pronounced shift towards gregariousness (Fig. 6A). Combining 5HTP with either of these two brief stimulus regimes, however, promoted increased gregariousness. There was little difference in walking speed under all six treatments, as even the saline-treated controls walk considerably more rapidly (1.4 cm s^−1^; 0–1.8 cm s^−1^) than the solitarious model population. Median resting time proportion (Fig. 6C) was 92% (80–99%) for the saline controls and locusts injected with 5HTP only (76–96%) but decreased to median values of 64% (52–82%) for locusts crowded when treated with 5HTP and 76% (68–87%) for locusts receiving tactile stimulation and 5HTP. The median grooming frequency (Fig. 6D) was zero for all groups. Only in the treatments where 5HTP was combined with a gregarizing stimulus was there evidence for some locusts having an increased proclivity to groom during the assay.

Locusts that had only been injected with saline or 5HTP rarely approached the stimulus group (median 0 s (0–0 s) time spent at the stimulus end), and across these two treatments only 10.7% of locusts did so (Fig. 6E). 31.3% of locusts that had received both the 5HTP injection and 30 min of crowding approached the stimulus group; a significant increase from controls (Table 8). Just 30 min of tactile stimulation on its own was sufficient to induce 57.9% of locusts to approach the stimulus group, whereas combining this treatment with an injection of 5HTP led to the great majority of locusts (77.8%) spending at least some time in close proximity with other locusts (Fig. 6E and insets; Table 8).

#### 3.3.2 The effect of inhibiting the synthesis or action of serotonin

**Fig. 7:**
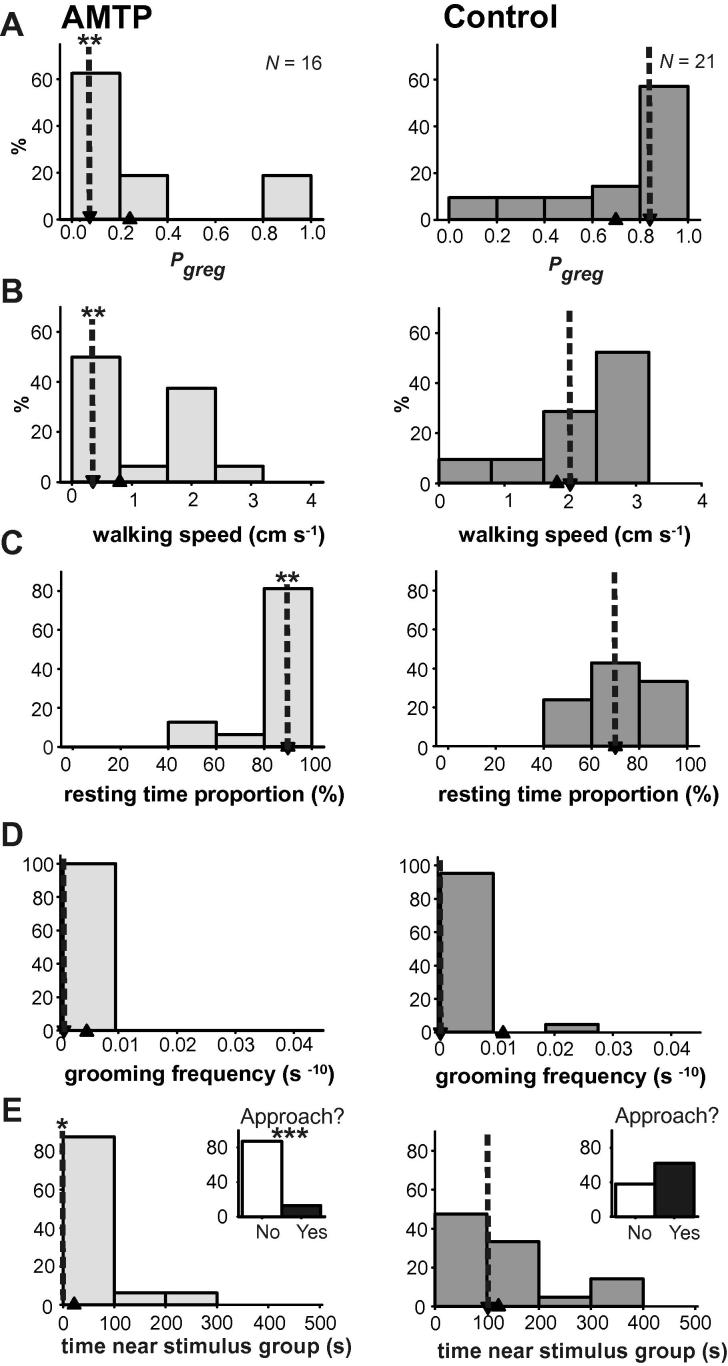
The effect of the serotonin synthesis inhibitor α-methyl tryptophan (AMTP; left) or a saline control (right) on the acquisition of gregarious behaviour in locusts subjected to 2 h of tactile stimulation to the hind femora. Data are histograms showing *P*_greg_ (A) and the individual behaviours used to generate it: walking speed (B), resting time proportion (C), grooming frequency (D) and time spent adjacent to the stimulus group of locusts in the arena (E). The inset graphs show the frequency of locusts approaching the stimulus group at least once during the 500 s observation period. Median values are indicated by down-pointing arrow heads with dotted lines and mean values by up-pointing arrow heads. ^∗^Indicates difference between control (saline) and AMTP treatment is *P* < 0.05; ^∗∗^indicates *P* < 0.01 and ^∗∗∗^*P* < 0.001; see also Table 9.

We demonstrated that interfering with the synthesis of serotonin using α-methyl tryptophan (AMTP) prevents the acquisition of gregarious behaviour when locusts are presented with otherwise gregarizing stimuli (Anstey et al., 2009). In this experiment locusts were pre-treated by repeated 40 μl injections of 0.1 mM AMTP or a locust saline control 5 days, 3 days and 1 day before the experiment, and on the day of the experiment itself, when locusts also were subjected to 2 h of tactile stimulation on a hind femur before being observed in the arena. Like 5HTP, AMTP injected systemically has a behavioural effect in Orthoptera (Stevenson et al., 2000). AMTP-treated locusts failed to gregarize, whereas the saline treated controls underwent considerable behavioural change (Fig. 7A). Fig. 7B–E presents the breakdown of the behavioural components used to generate these *P*_greg_ values. Three of the four behaviours were significantly different between AMTP- and saline-treated locusts, with only grooming frequency failing to show a statistical difference (Table 9). Median walking speed in AMTP treated locusts (Fig. 7B) remained similar to that of the solitarious model population at 0.6 cm s^−1^ (0–1.6 cm s^−1^), whereas in control locusts it increased to 2.0 cm s^−1^ (1.5–2.2 cm s^−1^), faster than the gregarious model population, but similar to other experiments where locusts had received 1–2 h of gregarizing stimulation. The proportion of time spent motionless (Fig. 7C) was 93% (87–99%) in AMTP treated locusts, again similar to the solitarious model population, but in control locusts it had decreased to 73 (63–84%) which was still more than in the long-term gregarious model population (61%; 51–72%). Grooming (Fig. 7D) was a rare occurrence, with a median frequency of 0 in both treatment groups. AMTP-treated locust avoided the other locusts in the arena (Fig. 7E), spending a median time of 0 s (0–0 s) near them, and only 2 out of 16 locusts (12.5%) approached at all. By contrast 13 out of 21 control locusts (62%) approached the stimulus group. The frequency of experimental locusts approaching the stimulus group was significantly different between the AMTP and control cohorts (*G*-test; *G* = 31.1, *P* < 0.001), and the proportion of control locusts spending time next to the stimulus group was indistinguishable from that of the long-term gregarious model population (*G*-test; *G* = 0.087, *P* = 0.768).

**Fig. 8:**
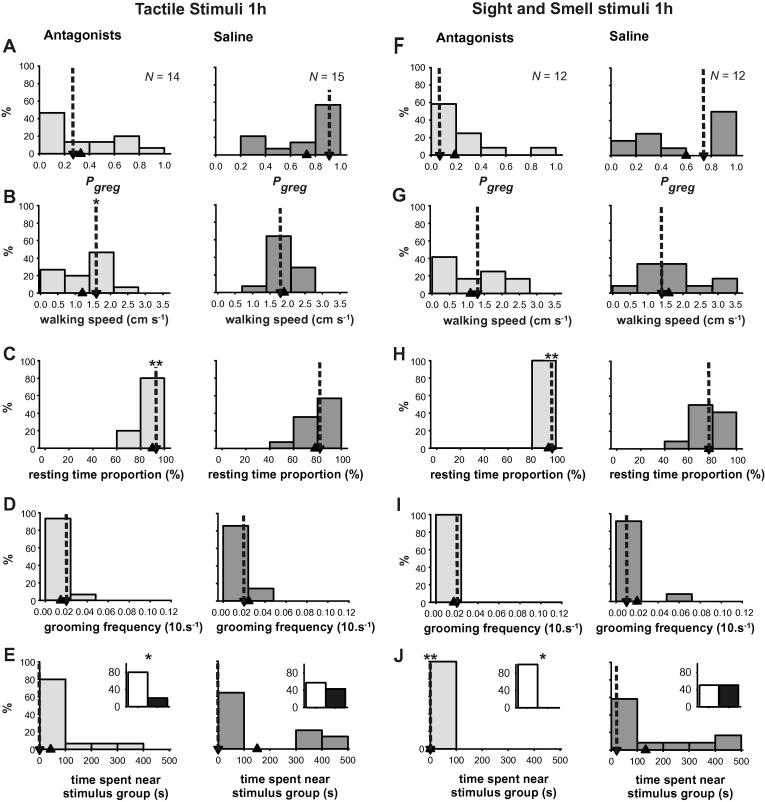
The effect of a cocktail of two serotonin receptor antagonists 1 mM ketanserin and 1 mM methiothepin (light grey) or saline controls (dark grey) injected into the thoracic ganglia on the acquisition of gregarious behaviour in locusts either given tactile stimulation to a hind femur for 1 h (left two columns A-E) or subjected to the sight and smell of other locusts (right two columns F-J) for 1 h. Data are histograms showing *P*_greg_ (A, F) and the individual behaviours used to generate it: walking speed (B, G), resting time proportion (C, H), grooming frequency (D, I) and time spent adjacent to the stimulus group of locusts in the arena (E, J). The inset graphs show the frequency of locusts approaching the stimulus group at least once during the 500 s observation period. Median values are indicated by down-pointing arrow heads with dotted lines and mean values by up-pointing arrow heads. ^∗^Indicates difference between control (saline only) and antagonist treated locusts is *P* < 0.05 and ^∗∗^indicates *P* < 0.01; see also Table 10.

The final way in which we interfered with the action of serotonin was to use a mixture of two serotonin receptor antagonists, 1 mM ketanserin and 1 mM methiothepin in locust saline, that interfere with the binding of serotonin onto its receptors (Anstey et al., 2009). This mixture, or a saline control were injected directly into the meso- and metathoracic ganglia of solitarious locusts, which were then subjected to 1 h of gregarizing stimuli, either tactile stimulation of a hind femur (Fig. 8A–E) or exposure to the sight and smell of the gregarious locust colony (Fig. 8F–J). The receptor antagonists prevented the acquisition of gregarious behaviour through both the tactile (Fig. 8A) and visual/olfactory pathways (Fig. 8B), whereas control animals undergoing either gregarizing treatment manifested a marked shift towards gregarious behaviour. At the level of the individual behaviours, walking speed was significantly higher in control locusts given the mechanosensory stimulus (1.8 cm s^−1^; 1.5–2.1 cm s^−1^) than in similarly treated locusts given the antagonists (1.6 cm s^−1^; 0–1.8 cm s^−1^; Table 10) but not for those given the olfactory and visual stimulus (1.4 cm s^−1^; 1.2–2 cm s^−1^ vs. 1.3 cm s^−1^; 0–1.8 cm s^−1^). The proportion of time spent motionless (Fig. 8C) was significantly less in control than in antagonist-treated locusts under both gregarizing conditions (Table 10; 82; 68–85% vs. 93%; 83–95% for mechanosensory treated locusts; 77%; 67–91% vs. 97%; 90–98% for olfactory + visual treated locusts). Grooming frequency (Fig. 8D) was again not significantly different between control and antagonist-treated locusts under either gregarizing regime (Table 10; medians’ range: 0.001–0.002 s^−1^). The median time spent near the stimulus group of locusts for the locusts given the mechanosensory gregarizing stimuli was 0 s for both the control (0 s; 0–358 s) and antagonist-treated (0 s; 0–0 s) groups. Control locusts subjected to the olfactory + visual stimulus, however, spent more time near the stimulus group (21.5 s; 0–293 s) than antagonist treated locusts (0 s; 0–0 s; Fig. 8E).When the proportion of locusts approaching or avoiding the stimulus group was considered, however (insets Fig. 8E), then the control locusts given either kind of gregarizing stimulus were significantly more likely to approach other locusts than the antagonist-treated locusts. For the mechanosensory-gregarized locusts, 6 out of 14 control locusts approached compared to 3 out of 15 antagonist-treated (*G*-test, *G* = 3.762, *P* = 0.05). For the olfactory + visually gregarized locusts, 6 out of 12 locusts approached the stimulus group, compared to 0 out of 12 controls (*G*-test using the 3 out of 15 of other antagonist-treated group as the expected frequency since expected frequencies of 0 cannot be computed, *G* = 5.355, *P* = 0.021). The proportions of control locusts that approached the stimulus group following 1 h of gregarising stimulation (42% and 50% for mechanosensory and olfactory + visual, respectively) are similar to the 47% observed after 1 h of either crowding or mechanosensory stimulation recorded in the time-course of gregarization experiments reported above.

## 4 Discussion

Our laboratory populations of long-term crowd-reared and three generation isolated-reared locusts can be separated convincingly on the basis of their behaviour in the assay arena. The behaviours by which they are most readily separable in the assay are pertinent to their behaviour in their natural environments. Consistent with their reliance on crypsis and inconspicuousness, isolated-reared locusts moved slowly and infrequently; furthermore, they mostly avoided the end of the arena adjacent to the stimulus group of locusts, with only 15% of them approaching at all during the assay. Crowd-reared locusts by contrast were highly active, walked rapidly and often, and 65% of them spent some time in close proximity to the stimulus group. These frequencies of approach to other locusts for each phase are remarkably similar to those obtained by Tanaka and Nishide (2013) who report values ranging from 10% to 19% for solitarious locusts and from 53% to 67% for gregarious locusts (measurements taken from Figs. 2 and 3 in their paper), despite differences in the details of the design of the arena and in the presentation of the stimulus group of locusts.

The observation that only two-thirds of gregarious locusts closely approach other locusts in either arena design is perhaps surprising, but presumably reflects the inherent constraints of an arena-style assay. Locust groups in the field are not static, but generally mobile, in particular during the day when they March collectively in migratory bands. The stimulus group of locusts in the arena are held in a restricted space and are unable to engage in marching or other group behaviours that may provide a stronger or more salient stimulus for a test locust introduced into the arena. The main compartment of the arena is also relatively small and a gregarious experimental locust introduced into this space may be sufficiently close to the stimulus group at any location within it to perceive itself to be part of the group. Marching occurs within a range of spacing between adjacent locusts in the migratory band and it is not the case that gregarious locusts simply move to be as close as possible to each other at all times (Buhl et al., 2011). The pronounced tendency for solitarious locusts to move to the opposite end of the arena and stay as far as possible from the stimulus group suggests that stimuli from other locusts are detectable and can elicit a behavioural response across the entire length of the arena. Our assay is not designed to provide an accurate proxy for locust behaviour in the field but to provide a means of behaviourally placing a test locust along the multidimensional trajectory between the two extreme phases; hence the use of substantial model populations of known solitarious and gregarious locusts to provide a detailed picture of how locusts of each phase may be expected to behave when placed in this new environment.

### 4.1 The time course of gregarization

Isolated-reared locusts undergo a dramatic change in behaviour within just a few hours of forced crowding, which encompasses all the behaviours used to generate the logistic regression model, both locomotion-related and attraction to the stimulus group. When locusts were forced into a crowd all four behavioural characters were significantly different from solitarious values within 1 h. When locusts were given a focal mechanosensory gregarizing stimulus to a hind femur, three of the four behaviours were significantly different within 1 h, with only grooming frequency not reaching significance until after 4 h of stimulation. After receiving gregarizing stimuli for 4 h, behaviours of initially solitarious phase individuals were closer in value to those of long-term gregarious locusts than of the solitarious locusts. The median proportion of time spent resting was 82–100%, median walking speed was 86–194% and time spent near the stimulus group was 53–145% of the values associated with long-term gregarious locusts. The proportion of locusts approaching the stimulus group at least once after 4 h of treatment was 85–92% of that of long-term gregarious locusts and statistically indistinguishable. Only grooming frequency was somewhat lower in the crowded or mechanosensorily stimulated locusts, but this probably reflects the rare occurrence of grooming. The median grooming frequency in the gregarious model population of 0.006 s^−1^ represents just three grooming events in the 500 s observation time. Whereas differences in the frequency of low-occurrence events can be detected in the large populations used to generate the logistic regression model, in the smaller sample sizes typically used in experimental treatments such differences are likely to be missed through stochastic variation. In summary, the locusts used in these experiments had undergone the greater part of the transformation to behavioural gregariousness after just 4 h of receiving gregarizing stimuli, whether considered at the level of individual activity and position-related behaviours or at the level of the aggregate behavioural index *P*_greg_.

In this meta-analysis we have also measured gregariousness purely on the basis of the likelihood of approach to other locusts, using a methodology similar to that of Tanaka and Nishide (2013). This analysis leads to the same conclusion first reached by Roessingh and Simpson (1994) and reported in many papers since: that behavioural gregarization in our laboratory populations of *S.* *gregaria* proceeds rapidly and occurs in the space of a few hours. We suggest that the much slower time course of gregarization reported by Tanaka and Nishide (2013) arises from biological differences in the populations of locusts studied, not from methodological differences in the way in which behaviour is measured. Substantial genetic variation between locust colonies has been demonstrated (Berthier et al., 2010) and this has the potential to confound observations arising from different laboratories using different strains and rearing protocols.

The rapid time course of behavioural gregarization in *S.* *gregaria* has been noted by other researchers. Ellis (1959) demonstrated that third instar nymphs of *S. gregaria* “learned to aggregate socially” within 7 h of either forced crowding, or mechanical stimulation with fine wires that kept the animals constantly agitated (see Fig. 7 therein). Ellis (1959) used overall aggregation as a measure of gregarization utilizing a toroidal arena in which the floor had been divided into 30 sections of equal size. She then counted the animals that had settled in each section at various time points, and tested whether these counts differed significantly from a random Poisson distribution. Ellis also took further behavioural observations of individual nymphs within the first and fourth hours after encountering other locusts for the first time. After 1 h, 74.3% of animals either jumped away from conspecifics or moved back a few steps, with the rest sitting still (and in one case twirling antennae and hind-leg kicking, which Ellis designated as a gregarious characteristic). This pattern had shifted dramatically after 4 h, with only 13.4% of nymphs moving away from conspecifics, and 31.1% displaying the gregarious kicking and antennal twirling behaviours (see Table 5 therein). Ellis (1963) later investigated social aggregation using a precursor to the ‘stimulus group’ employed by (and since) Roessingh et al. (1993). Ellis (1963) tethered groups of gregarious hopper ‘decoys’ to the floor of a circular arena, and recorded the amount of time that test locusts spent within 6 mm of these conspecifics. Solitarious locusts spent significantly less time near the decoys over a 30 min test period than animals that had been crowded with other locusts for 4 h (236.3 ± 52.4 s vs. 522.3 ± 63.3 s; *P* < 0.01), while these “trained” locusts were indistinguishable from long-term gregarious insects (522.3 ± 63.3 s vs. 535.2 ± 68.9 s; N.S.). Gillett (1973) showed a significant increase in clustering (into groups of three or more locusts) in solitarious second-instar nymphs over a 2 h crowding period, though she also noted a decrease in clustering in gregarious conspecifics; she noted that this pattern was likely due to the locusts being so active that they rarely settled into static groups. Heifetz et al. (1996) also demonstrated a marked increase in gregarious behaviour after 6 h of crowding in groups of 10, in fourth instar nymphs that had previously been isolated for 3 days. However, this study used a composite measure of phase state derived from discriminant analysis, and raw data for individual activity- and social interaction-based behaviours were not presented. It is therefore not possible to determine which particular behavioural characters contributed to the reported change in phase state.

### 4.2 The effect of changes in the expression of single behaviours on P_greg_

**Fig. 9:**
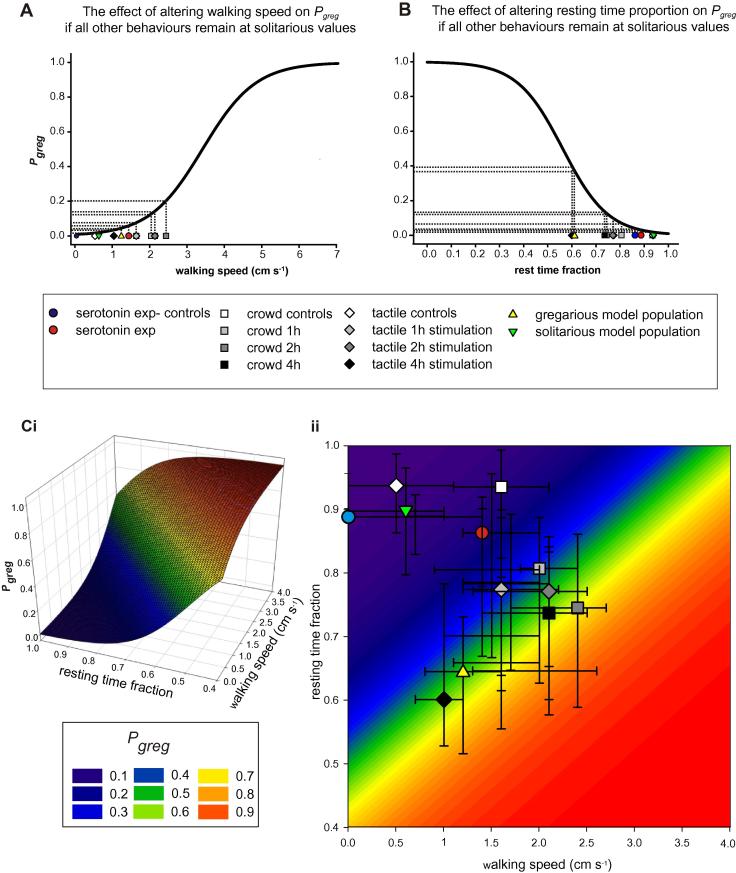
Simulations of the effect of systematically increasing walking speed (A), resting time proportion (B) or both together (C), whilst the other behaviours in the logistic equation are held at median solitarious values. The symbols plotted over the plots show the recorded median values for walking speed (A) and rest time fraction (B) recorded for each experiment. Both walking speed and rest time fraction have to be pushed into simulated values well above those observed in real locusts in order to produce *P*_greg_ values in the gregarious domain (>0.5). (Ci) Systematically altering both walking speed and resting time fraction produces a 3-dimensional contour of possible *P*_greg_ values. This is plotted as a contour map in (Cii) with the median and interquartile range of observed walking speeds and rest time proportions observed in locusts during the experiments overlaid.

Given that *P*_greg_ of solitarious locusts changed dramatically within the first few hours of crowding or experimental treatment, the individual behaviours contributing to *P*_greg_ must necessarily have changed over the same time course; but could this change be driven primarily by change in just one of the behaviours as suggested by Tanaka and Nishide (2013)? Walking speed for example increased to values above those of long-term gregarious locusts in the early stages of crowding or tactile stimulation. In locusts given tactile stimulation, walking speed decreased again after 4 h of stimulation, to a value just below that characteristic of long-term gregarious locusts, whereas in crowded locusts walking speed was still elevated. How much does this overdriven walking speed contribute to the value of *P*_greg_ obtained in the early stages of gregarization? To answer this question we simulated the change in *P*_greg_ that results from systematically increasing walking speed whilst keeping all other behaviours in the logistic equation at their solitarious values (Fig. 9A). Obviously, increasing the walking speed increases *P*_greg_ and this by itself will eventually lead to a *P*_greg_ approaching 1; but to increase *P*_greg_ only as high as 0.5, locusts would have to walk at 3.4 cm s^−1^, 42% faster than the fastest observed median walking speed (after 2 h crowding). The walking speeds associated with each experiment are plotted beneath the curve in Fig. 9A and it is clear that by themselves they have only a small effect on *P*_greg_.

The other locomotion-related measure in our model is the proportion of time spent resting. We determined its individual effect on *P*_greg_ in the same way as for of walking speed (Fig. 9B). A decrease in time resting results in an increase in *P*_greg_, hence the curvature of the graph is reversed relative to Fig. 9A. Resting time proportion across the range observed in the experimental locusts influenced *P*_greg_ more strongly than the natural range of walking speed, but even here, the lowest resting time proportions observed (in the gregarious model population and locusts given tactile stimulation for 4 h) would by themselves only give rise to *P*_greg_ values < 0.4 in the absence of other behavioural changes.

Finally, we systematically altered both walking speed and resting time proportion to produce a landscape of possible *P*_greg_ values associated purely with changes in locomotor activity (Fig. 9Ci). We then plotted the median and interquartile ranges of the observed resting time proportions and walking speed for all the treatments over the contour map of the simulation (Fig. 9Cii). Unsurprisingly, the combination of a high walking speed and low resting time proportion yields a higher value of *P*_greg_ than either gregarious character on its own, but even here the highest median *P*_greg_ obtainable for real data in the absence of position or grooming frequency was 0.65 (for 2 h crowded locusts, Fig. 9Cii). It is possible for a locust to have quite a high value of *P*_greg_ without going towards the stimulus group, but to obtain values above 0.7 in most cases requires that the locust approaches the stimulus group of locusts.

A weakness in relying solely on the proximity of test locusts to other locusts as a measure of gregariousness is that approximately a third of long-term crowded locusts fail to approach the stimulus group (Fig. 1C). Therefore when using proximity as the only indicator of behavioural phase there is an expectation that under all experimental conditions a third of behaviourally gregarious locusts will be mis-classified. By contrast, logistic regression analysis incorporates both the direct and correlated effects of multiple behaviours comprising the behavioural phenotype. A key advantage of the logistic regression approach based on multiple behaviours is that it is not dependent on the high degree of stochastic variation in position within the arena and that a quantitative value can be given to the similarity or otherwise of individual observations to the model populations.

### 4.3 The effect of serotonin on behavioural phase in S. gregaria

Establishing a functional role for a neurochemical in behaviour requires multiple lines of evidence, which is why we used a variety of pharmacological agents (Anstey et al., 2009) to establish the critical role of serotonin in the early stages of behavioural gregarization. Breaking down *P*_greg_ into its behavioural constituents as we have now done (Figs. 4–8) shows that serotonin affects all locomotion- and position-related behaviours in the model. Serotonin applied directly to the thoracic nervous system in an appropriate manner that enables it to cross the blood–brain barrier, or systemic injections of a serotonin precursor that crosses the barrier unaided not only increased activity, but also the propensity of locusts to move towards their conspecifics. Moreover, blocking the action of serotonin or inhibiting its synthesis not only decreased activity, but also resulted in treated locusts avoiding the stimulus group. Given the clear evidence that even 1 h of gregarizing treatment increases the frequency of locusts approaching the stimulus group, it would be expected that locusts given serotonin inhibitors would still approach the stimulus group with the same frequency as controls if the effect of serotonin was confined to activity only. It is clear that enhancing serotonin signalling and increasing its availability not only increases locomotor activity, but also causes treated locusts to approach conspecifics rather than avoid them.

### 4.4 The role of serotonin in phase change in other species of locust

Guo et al. (2013) working on the role of serotonin during behavioural phase change in the migratory locust (*Locusta migratoria*) referred to a ‘controversial role of serotonin in regulating phase change’, since their data point to a role for serotonin in regulating solitarization and an ambiguous or negative role in gregarization in this species. Ma et al. (2011) provided evidence that dopamine, another biogenic amine, is important for the expression of gregarious behaviour in *L. migratoria*, but that serotonin may facilitate the transition from solitarious to gregarious behaviour. There are several reasons why the apparent role of serotonin may be different in *L. migratoria* and *S. gregaria*.

Guo et al. (2013) reported no significant change in serotonin titres in the brain over a 32 h period of crowding 4th instar *L. migratoria* nymphs, but *L. migratoria* does not undergo behavioural gregarization within this period. In an earlier paper, Guo et al. (2011) presented a time course of behavioural change over 64 h of crowding, and even though there is a small increase in *P*_greg_ (and its constituent behaviours) within this period, the maximum median value of *P*_greg_ achieved is only ∼0.3 after 32 h, but by 64 h *P*_greg_ had dropped again to a median value of ∼0.2. In Guo et al., 2013, the converse measure, *P*_sol_ is used (1 − *P*_greg_) and here after 32 h crowding locusts had a median *P*_greg_ of <0.4. Even though these small changes were significant, the behavioural transformation was evidently far from complete and as yet it is unclear how long full behavioural gregarization takes in *L. migratoria*. Even in the slowly gregarizing *S. gregaria* population used by Tanaka and Nishide (2013) approximately 50% of the change to gregarious values of attraction is achieved with 48 h and complete transition within 5 days. In *S. gregaria*, the increase in serotonin only occurs during the critical period of behavioural transformation (Rogers et al., 2004; Anstey et al., 2009): nymphs crowded for one instar have serotonin titres similar to those of long-term gregarious locusts, which are slightly *lower* than those of long-term gregarious nymphs (Rogers et al., 2004). As such Serotonin is not a marker of gregariousness *per se* in *S. gregaria*, but facilitates the *transition* from solitarious to gregarious behaviour and because of this only has a small window of activity (Burrows et al., 2011; Ott et al., 2012; Rogers, 2014). Manipulation of protein kinase A (PKA) signalling, which is a downstream target of serotonin signalling within the *Schistocerca* CNS during gregarization, had no effect on the behaviour of the fully established gregarious phase, but prevented the transition from solitarious to gregarious behaviour (Ott et al., 2012).

Guo et al. (2013) analysed the effect of manipulating the 5HT_2_ receptor pathway on behavioural change in *L. migratoria* and they concluded that it had no role in promoting gregarization, or even inhibited the process. 5HT_2_ receptors act primarily through activation of phospholipase C (PLC) to generate the second messenger inositol trisphosphate (IP3) (Leysen, 2004; Spitzer et al., 2008). Behavioural gregarization in *S. gregaria* is mediated via a PKA-dependent pathway (Ott et al., 2012), which is activated by cAMP. This strongly suggests that another serotonin receptor subtype, one that is positively coupled to adenyl cyclase, such as the 5HT_7_ receptor for example (Vleugels et al., 2013), is implicated in behavioural gregarization in this species. We would therefore not expect that activation of the 5HT_2_ receptor would promote gregarization in *S. gregaria*.

If the role of serotonin during phase change is to facilitate behavioural/neuronal plasticity during gregarization rather than in the expression of gregarious behaviour itself, then it could also have a role in the reverse process of solitarization. The establishment of the full gregarious phase is accompanied by extensive changes in gene expression (Kang et al., 2004; Badisco et al., 2011; Guo et al., 2011) so that signalling systems are acting on a different underlying physiology in the two phases. Furthermore, the same transmitter could act through different receptor subtypes and/or target different regions of the CNS in the two processes.

The process of solitarization appears to be very different in *S. gregaria* and *L. migratoria*. In *S. gregaria*, there is a partial loss of gregarious behaviour within a few hours, but this is followed by a semi-gregarious behavioural state that is stable for at least one week (Roessingh and Simpson, 1994). In *L. migratoria*, isolation of long-term gregarious locusts leads to a complete loss of gregarious character within just a few hours (Guo et al., 2011). With respect to the rate of change, therefore, gregarization and solitarization have opposite characteristics in the two species: rapid gregarization and slow solitarization in *S. gregaria*; slow gregarization and rapid solitarization in *L. migratoria*. Differences in the mechanisms mediating these behavioural transformations in the two species may therefore be expected. Isolation of long-term gregarious *S. gregaria* is accompanied in a surge of serotonin, dopamine and octopamine in the first 24 h of separation (Rogers et al., 2004), but much work remains to be done on the mechanism driving behavioural solitarization in this species.

Part of the reason underlying the differences in behavioural phase change between *S. gregaria* and *L. migratoria* is that they belong to different subfamilies of the Acrididae, the Cyrtacanthacridinae and Oedipodinae, respectively. Two reports (Fries et al., 2007; Vickery, 1989) place the separation of the Oedipodinae from other grasshopper subfamilies as occurring in the Cretaceous period (∼90 million years ago). Phase polyphenism is a rare occurrence within the Acrididae, with only approximately 20 out of a total of 11,000 named species showing the full suite of behavioural, morphological and physiological changes and these are found scattered across several subfamilies (Song, 2011; Pener and Simpson, 2009). Phase change in distantly related Acridids is therefore almost certainly a homoplasy in response to similar environmental conditions. Close similarities in mechanism may be expected within clades where several closely related species show either full or partial phase change (e.g. within the genus *Schistocerca* and wider Cyrtacanthacridinae; Song and Wenzel, 2008). Any similarities between more distantly related locust species will likely depend upon the common recruitment of general mechanisms of neuronal plasticity and regulation of social interaction that have been evolutionarily conserved across the wider animal kingdom, rather than deriving from a shared evolutionary origin of phase change itself. As such considerable difference in the details of mechanisms must be expected. Even in the unlikely event that some form of phase change is basal to the Acrididae, which has subsequently been lost in the great majority of taxa, the long separation of the Cyrtacanthacridinae from the Oedipodinae would allow for substantial divergence in the mechanisms of phase change to occur.

## Figures and Tables

**Table 1:** Parameters in the logistic regression model. Significant results are in bold. See also Fig. 1.

	*B*	S.E.	Wald	d.f.	*P*
Walking speed	1.405	.304	21.430	1	**<0.001**
Resting time fraction	−10.569	1.440	53.886	1	**<0.001**
Grooming frequency	496.605	77.336	41.234	1	**<0.001**
Time at stimulus end	0.003	.001	5.016	1	**0.025**
Constant	5.192	1.099	22.340	1	**<0.001**

**Table 2:** Statistical differences between the four behavioural parameters used in the logistic regression between the solitarious and gregarious locusts that comprised the model populations. Significant results are in bold. See also Fig. 1.

	Walking speed	Resting time fraction	Total grooming frequency	Time at stimulus end
Mann–Whitney *U*	9535.5	2955.5	7085.0	9552.5
*Z*	−8.66	−14.508	−11.78	−9.70
*P*	**<0.001**	**<0.001**	**<0.001**	**<0.001**

**Table 3:** Statistical differences tested using the Mann–Whitney test for the four behavioural characters used in the logistic regression between control (solitarious) locusts and locusts that were crowded for 1, 2 and 4 h respectively. Significant results are shown in bold; See also Fig. 2.

0 h (*N* = 25)	1 h crowding (*N* = 40)			2 h crowding (*N* = 47)			4 h crowding (*N* = 49)		
	*U*	*Z*	*P*	*U*	*Z*	*P*	*U*	*Z*	*P*
Walking speed	341	−2.146	**0.032**	361.5	−2.676	**0.007**	476.	−1.563	0.118
Resting time fraction	173.5	−4.406	**<0.001**	115.5	−5.584	**<0.001**	169	−5.071	**<0.001**
Total grooming frequency	412.5	−2.194	**0.028**	462.5	−2.460	**0.014**	512	−2.121	**0.034**
Time at stimulus end	362.5	−2.170	**0.030**	407.0	−2.456	**0.014**	392	−2.811	**0.005**

**Table 4:** Statistical differences tested using the Mann–Whitney test for the four behavioural characters used in the logistic regression between control (solitarious) locusts and locusts that have received mechanosensory stimuli to a hind femur for 1, 2 and 4 h respectively. Significant results are shown in bold; See also Fig. 3.

0 h (*N* = 35)	1 h mechanosensory (*N* = 34)			2 h mechanosensory (*N* = 49)			4 h mechanosensory (*N* = 60)		
	*U*	*Z*	*P*	*U*	*Z*	*P*	*U*	*Z*	*P*
Walking speed	228	−4.452	**<0.001**	254.500	−5.509	**<0.001**	710.500	−2.630	**0.009**
Resting time fraction	275	−3.844	**<0.001**	390.500	−4.242	**<0.001**	306.000	−5.742	**<0.001**
Time at stimulus end	456	−1.962	**0.050**	542.500	−3.169	**0.002**	603.000	−3.751	**<0.001**
Total grooming frequency	528	−1.447	0.148	767.500	−1.099	0.272	667.000	−3.370	**0.001**

**Table 5:** The results of Mann Witney tests on the individual behaviours used in the logistic regression between locusts that received the serotonin topically applied to the thoracic ganglia and those receiving a saline control. Significant results are shown in bold; see also Fig. 4.

*N* = 18 5HT treated *N* = 12 control treated	Walking speed	Resting time fraction	Time at stimulus end	Total grooming frequency
Mann–Whitney *U*	53.5	91.0	77.0	85.
*Z*	−2.329	**−**0.720	−1.564	−1.108
*P*	**0.020**	0.472	0.118	0.268

**Table 6:** Serotonin was ineffective at eliciting behavioural gregarization when injected into the haemocoel. The results of Mann–Whitney tests on values of *P*_greg_ and on each of the four individual behaviours used in the logistic regression model between locusts injected with 1 mM serotonin into the haemolymph or a saline control. See also Fig. 5.

*N* = 70 5HT treated *N* = 26 control	*P*_greg_	Walking speed	Resting time fraction	Total grooming frequency	Time at stimulus end
Mann–Whitney *U*	727	754	744	896	784
*Z*	−1.509	−1.302	−1.369	−0.174	−1.366
*P*	0.131	0.193	0.171	0.862	0.172

**Table 7:** The results of Kruskal–Wallis tests on the four behavioural parameters used in the logistic regression analysis in experiment where locusts received the serotonin precursor or a saline control, either without further stimulation or having been crowded for a period of 30 min or receiving mechanosensory stimulation on the hind femur for 30 min. Significant results are shown in bold. See also Fig. 6.

	Walking speed	Resting time fraction	Total grooming frequency	Time at stimulus end
Chi-Square	7.77	20.462	3.574	38.108
*df*	5	5	5	5
*P*	0.169	**0.001**	0.612	**<0.001**

**Table 8:** Results of *G-*tests on the frequency of locusts approaching the stimulus group after pre-treatment with either the serotonin precursor 5-hydroxy tryptophan (5HTP) or a control saline injection and then receiving no further treatment, or experiencing 30 min of crowding with other locusts or 30 min of tactile stimulation to a hind femur. Significant results are shown in bold; see also Fig. 6.

Saline injection only *N* = 14	Saline vs. 5HTP only	Saline vs. crowding only	Saline vs. 5HTP + crowding	Saline vs. touch only	Saline vs. 5HTP + touch
*N*	17	16	16	19	27
*G*	0.464	0.575	8.146	33.38	83.13
*P*	0.496	0.448	**0.004**	**<0.001**	**<0.001**

**Table 9:** Statistical differences for the four behavioural parameters used in the logistic regression between locusts that received the serotonin synthesis inhibitor AMTP or a saline control over a period of several days before receiving tactile stimulation on the hind femur for 2 h. Significant results are shown in bold; See also Fig. 7.

*N* = 16 AMTP treated *N* = 21 control treated	Walking speed	Resting time fraction	Total grooming frequency	Time at stimulus end
Mann–Whitney *U*	69.5	84.0	164.0	93.0
*Z*	−3.052	−2.897	−0.163	−2.301
*P*	**0.002**	**0.004**	0.870	**0.021**

**Table 10:** Statistical differences for the four behavioural parameters used in the logistic regression between locusts that received a cocktail of two serotonin receptor antagonists or a saline control before receiving either tactile stimulation on a hind femur for 1 h or being subjected to the sight and smell of the long-term gregarious colony. Significant results are shown in bold; see also Fig. 8.

	Walking speed	Resting time fraction	Total grooming frequency	Time at stimulus end
*N* = 15 antagonist treated *N* = 14 control treated	Mechanosensory stimulation			
Mann–Whitney *U*	55.0	42.5	73.5	76.0
*Z*	−2.185	−2.730	−1.476	−1.543
*P*	**0.029**	**0.006**	0.140	**0.123**

*N* = 12 antagonist treated *N* = 12 control treated	Visual + Olfactory stimulation			
Mann–Whitney *U*	54	25	63.5	36
*Z*	−1.044	−2.714	−0.525	−2.732
*P*	0.297	**0.007**	0.6	**0.006**
